# Supplementary figures and images for: A Versatile and Robust Serine Protease Inhibitor Scaffold from Actinia tenebrosa
Source: Mar Drugs. 2019 Dec 12;17(12):701. doi: 10.3390/md17120701 (PMC6950308; doi:10.3390/md17120701)

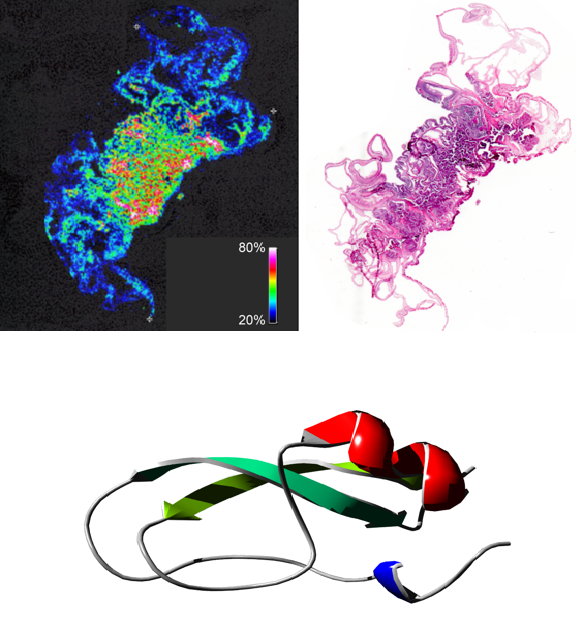

Supplement: Supplementary file 1 [file marinedrugs-17-00701-s001.jpg]
